# Ketamine Blocks Morphine-Induced Conditioned Place Preference and Anxiety-Like Behaviors in Mice

**DOI:** 10.3389/fnbeh.2020.00075

**Published:** 2020-05-21

**Authors:** Greer McKendrick, Hannah Garrett, Holly E. Jones, Dillon S. McDevitt, Sonakshi Sharma, Yuval Silberman, Nicholas M. Graziane

**Affiliations:** ^1^Neuroscience Graduate Program, Penn State College of Medicine, Hershey, PA, United States; ^2^Department of Anesthesiology and Perioperative Medicine, Penn State College of Medicine, Hershey, PA, United States; ^3^Summer Undergraduate Research Internship Program, Penn State College of Medicine, Hershey, PA, United States; ^4^Department of Neural and Behavioral Sciences, Penn State College of Medicine, Pennsylvania State University, Hershey, PA, United States; ^5^Departments of Anesthesiology and Perioperative Medicine and Pharmacology, Penn State College of Medicine, Hershey, PA, United States

**Keywords:** negative affect, morphine, conditioned place preference, anxiety, opioid use disorder, ketamine, psychedelics

## Abstract

Patients suffering from opioid use disorder often relapse during periods of abstinence, which is posited to be caused by negative affective states that drive motivated behaviors. Here, we explored whether conditioning mice with morphine in a conditioned place preference (CPP) training paradigm evoked anxiety-like behavior during morphine abstinence. To do this, mice were conditioned with morphine (10 mg/kg, i.p.) for 5 days. Twenty-four hours following conditioning, anxiety levels were tested by measuring time in the open arms of the elevated plus-maze. The next day, mice were placed in the three-compartment chamber to measure morphine-induced CPP. Our results show that following morphine conditioning, mice spent significantly less time in the open arm of the elevated plus-maze and expressed robust morphine CPP on CPP test day. Furthermore, we found that an acute treatment with (*R,S*)-ketamine (10 mg/kg, i.p.), a medication demonstrating promise for preventing anxiety-related phenotypes, 30 min before testing on post-conditioning day 1, increased time spent in the open arm of the elevated plus-maze in saline- and morphine-conditioned mice. Additionally, we found that the second injection of ketamine 30 min before CPP tests on post-conditioning day 2 prevented morphine-induced CPP, which lasted for up to 28 days post-conditioning. Furthermore, we found that conditioning mice with 10% (w/v) sucrose using an oral self-administration procedure did not evoke anxiety-like behavior, but elicited robust CPP, which was attenuated by ketamine treatment 30 min before CPP tests. Overall, our results suggest that the ketamine-induced block of morphine CPP may not be attributed solely to alleviating negative affective states, but potentially through impaired memory of morphine-context associations.

## Introduction

The motivation to continually seek and obtain addictive substances during periods of abstinence or recovery is caused, in part, by the necessity to avoid aversive internal states (Solomon and Corbit, 1978). Evidence for this comes from patients with substance use disorders who self-report urges and intentions to take drugs to avoid drug-withdrawal symptoms (O’Brien, 1975; Baker et al., 2004; Wikler, 2013) or to cope with negative affect (Perkins and Grobe, 1992; Zinser et al., 1992; Wetter et al., 1994; Cooney et al., 1997; Conklin and Perkins, 2005; Fox et al., 2007). For example, abstinence from morphine, a highly addictive opioid, facilitates increases in anxiety (Gold et al., 1978, 1979), which is a potential factor in continued drug use (Martins et al., 2012).

To better understand the mechanisms mediating drug-craving and subsequent relapse, preclinical models have been developed whereby drug-seeking behaviors are monitored in drug-exposed rodents. In the conditioned place preference (CPP) paradigm, a drug is paired with a context during conditioning. This is followed by a test day whereby the time spent in the drug-paired context is measured. This behavioral paradigm is a form of Pavlovian learning whereby injection of a drug (i.e., unconditioned stimulus) elicits a hedonic feeling of pleasure (i.e., unconditioned response), which, when paired with a context (neutral stimulus), invokes incentive value to the context (i.e., now a conditioned stimulus), thus driving a behavioral response to “seek” the context (conditioned response). This is similar to sign-tracking behaviors (Huston et al., 2013), which refer to a behavior that is directed toward a stimulus as a result of that stimulus becoming associated with a reward (Huys et al., 2014). Therefore, CPP provides a valuable tool used to understand how drugs of abuse become associated with environmental contexts, which is implicated in context-induced drug craving and relapse (O’Brien and Ternes, 1986; O’Brien et al., 1992). We have found that 5 days of morphine (10 mg/kg) conditioning elicits robust morphine CPP (Graziane et al., 2016; McDevitt and Graziane, 2019). However, it is unclear whether this “drug context-seeking” behavior is mediated by negative affective states. Additionally, it is unclear whether a subanesthetic dose of ketamine, an anxiolytic agent (Engin et al., 2009), blocks morphine-induced CPP by mitigating morphine-induced negative affective states.

Here, we attempt to investigate whether morphine conditioning in our CPP paradigm generates negative affect during morphine abstinence. Additionally, we investigate whether an acute, subanesthetic dose of (*R,S*)-ketamine before testing is sufficient to disrupt morphine-induced anxiety and/or morphine-induced CPP behaviors. Lastly, it has been shown that an acute administration of (*R,S*)-ketamine is sufficient to block the expression of morphine CPP (Suzuki et al., 2000). Here, we investigate whether this ketamine-induced block of morphine CPP, in our behavioral training paradigm, is mediated by the impairment of drug-context associations or by the attenuation of morphine-induced negative affective states.

## Materials and Methods

### Animals

All experiments were done following procedures approved by the Pennsylvania State University College of Medicine Institutional Animal Care and Use Committee. Male C57BL/6J mice aged 5–8 weeks were purchased from Jackson Labs (stock #000664; Bar Harbor, ME, USA), singly-housed, and maintained on a regular 12 h light/dark cycle (lights on 07:00, lights off 19:00) with *ad libitum* food and water. Mice were singly housed for the following reasons. First, we have reliably developed morphine conditioned place preference (CPP) in singly-housed mice (Graziane et al., 2016; McDevitt and Graziane, 2019). Second, evidence suggests that socially isolated rodents are more vulnerable to developing drug-context associations (Whitaker et al., 2013). In humans, social isolation increases vulnerability to substance use disorders (Newcomb and Bentler, 1988; Sinha, 2008), which often are accompanied by the development of drug-context associations (O’Brien and Ternes, 1986; O’Brien et al., 1992; Xue et al., 2012). Therefore, our studies are designed to model this patient population.

### Drugs

(−)-morphine sulfate pentahydrate was provided by the National Institute on Drug Abuse Drug Supply Program. Ketamine hydrochloride (racemic mixture of 50% *R*-ketamine and *S*-ketamine; Dechra Pharmaceuticals, Northwich, UK) was purchased from the Comparative Medicine Department at the Pennsylvania State University College of Medicine.

### Non-contingent Conditioned Place Preference

Conditioned place preference (CPP) chambers (Med Associates) were located in the mouse housing room and consisted of three distinct compartments separated by manual guillotine-style doors. Each compartment had distinct contextual characteristics: the middle (neutral) compartment (7.2 cm × 12.7 cm × 12.7 cm) had gray walls and gray plastic floor, while the choice compartments (16.8 cm × 12.7 cm × 12.7 cm, each) had either white walls and stainless steel mesh floor or black walls and stainless steel grid floor. All compartments were illuminated with dim light during use. Immediately following use, the entire preference chamber was cleaned thoroughly with a scent-free soap solution. Mouse locations, activity counts, and time spent in each compartment were collected *via* automated data-collection software (Med Associates) *via* infrared photo beam strips lining each compartment. Morphine administration was verified with the Straub tail response and enhanced locomotor activity (Bilbey et al., 1960; Graziane et al., 2016; McDevitt and Graziane, 2019).

#### Habituation

Mice were placed in the center compartment with free access to all three compartments for 20 min once a day for 2 days. Time spent (seconds) in each compartment was recorded.

#### Conditioning

Twenty-four hours after habituation, mice received 5 days conditioning training. Morphine-paired compartments were assigned based on the least preferred side (a biased approach; Tzschentke, 2007) calculated by averaging time spent in each compartment over the two habituation days. Similar to conditioning studies with alcohol (Gremel et al., 2006), we find that C57BL/6J mice will reliably develop morphine CPP using a biased approach. During conditioning, mice received an injection of saline and were placed into the most preferred compartment for 40 min. Six hours later, mice received an injection of saline (control group) or morphine (10 mg/kg, i.p.) and were placed into their least preferred compartment for 40 min (Koo et al., 2014; Graziane et al., 2016).

#### Post-conditioning

Forty-eight hours or 28 days after the last conditioning day, mice were placed in the 3-compartment chamber and allowed to move freely for 20 min. Our post-conditioning took place at a time point corresponding to 3 h before drug conditioning (e.g., morphine conditioning took place at 3 P.M., post-conditioning tests took place 2 or 28 days later at 12 P.M.). CPP scores were calculated as time spent in the drug-paired side minus the average time spent on the same side during preconditioning (Bohn et al., 2003). Activity counts are defined as any beam break within a current zone. This is inclusive of grooming, rearing, and lateral movements. Mice were treated with 0.9% saline (0.1 ml, i.p.) or with (*R,S*)-ketamine (10 mg/kg, i.p.) 30 min before the first CPP test. The dose of ketamine was selected based on preclinical data demonstrating that a 10 mg/kg dose of ketamine produces a maximal effect on morphine CPP (Suzuki et al., 2000) and produces plasma concentrations associated with subanesthetic ketamine doses capable of eliciting antidepressant effects in mice and humans (Zarate et al., 2012; Zanos et al., 2016).

### Sucrose Oral Self-administration Conditioned Place Preference

#### Habituation

Mice were placed in the center compartment with free access to all three compartments for 20 min once a day for 2 days. Time spent (seconds) in each compartment was recorded.

#### Conditioning

Drinking bottles were created as described in Freet et al. (2013). Briefly, we modified 10 ml serological pipettes by tapering both ends, placing a stainless-steel sipper tube (Ancare; OT-300) in one end and a silicone stopper (Thermo Fisher Scientific; 09-704-1D) in the other. Bottles were inserted into plastic holders that were then placed directly into CPP chambers (for chamber description, see “Non-contingent Conditioned Place Preference” section), where they were positioned so that the sipper was ~5 cm above the chamber floor. Pennsylvania State University Fabrication shop constructed plexiglass tops that were placed along the top of the 3-compartment apparatus and allowed for plastic bottle holders to be placed into chambers. Oral self-administration was recorded as the mL before and following all sessions. Similar to the i.p. CPP methodology, we utilized a biased approach in which the 10% sucrose (w/v) solution was placed in the least-preferred context. Twenty-four hours after habituation, mice underwent two 14 h overnight sessions (separated by 24 h), confined to the least preferred chamber on the first night (ON1) with access to water (control groups) or a 10% sucrose solution and confined to the most preferred side on the second night (ON2) with access to water. Mice then received 5 days of conditioning (C1–C5), where morning sessions consisted of 40 min in the most-preferred context with access to water. Six hours later, afternoon sessions consisted of 40 min in the least preferred context with access to water (control groups) or 10% sucrose solution.

#### Post-conditioning

Forty-eight hours or 21 days after the last conditioning day, mice were placed in the 3-compartment chamber and allowed to move freely for 20 min. Our post-conditioning took place at a time point corresponding to 3 h before drug conditioning (e.g., sucrose conditioning took place at 3 P.M., post-conditioning tests took place 2 or 21 days later at 12 P.M.). No bottles were present in the chambers on preference tests. CPP scores were calculated as time spent on the least preferred side on test day minus the average time spent on the same side during preconditioning (Bohn et al., 2003). Mice treated with (*R,S*)-ketamine (10 mg/kg, i.p.; water+ketamine and sucrose + ketamine groups) received injections 30 min before the first CPP test on post-conditioning day 2.

### Elevated Plus Maze

The elevated plus-maze, a well-established method to measure anxiety in rodents, was implemented to measure anxiety-like behavior (Pellow et al., 1985; Handley and McBlane, 1993; Dawson and Tricklebank, 1995). The elevated-plus maze for mice (Stoelting, Item #60140) was raised approximately 50 cm from the ground. The floor of the elevated portion of the maze was gray. Two opposite arms (35 × 5 cm each) of the maze were enclosed by a 15 cm high wall and the remaining two arms were “open.” A center space (5 cm^2^) between these four arms was also not enclosed. The elevated portion of the apparatus was cleaned thoroughly with a scent-free soap solution after each trial. Behavioral tests were performed in the animal housing room under ambient light of the light cycle.

Twenty-four hours after the last conditioning day in the CPP apparatus, mice were placed in the center space facing the open arm and allowed to explore the apparatus for 5 min before being placed back into their home cage (Grisel et al., 2008). Each trial was video recorded using a GoPro camera (Hero7 white) and analyzed by researchers blinded to the treatment condition of the mice. Time in the open arm was measured when the body of the mouse cleared the center space. Mice were treated with 0.9% saline (0.1 ml, i.p.) or ketamine (10 mg/kg, i.p.) 30 min before the elevated plus-maze test.

### Statistical Analysis

Statistical significance was assessed in GraphPad Prism software using a student’s *t*-test, one- or two-way ANOVA with Bonferroni’s correction for multiple comparisons as specified. *F* values for two-way ANOVA statistical comparisons represent interactions between variables unless stated otherwise. Two-tailed tests were performed for student’s *t*-test. For correlation analysis, Pearson’s correlation coefficient, and subsequent linear regression, were determined. *P* < 0.05 was considered to indicate a statistically significant difference.

## Results

### Morphine Conditioning Elicits Anxiety-Like Behaviors During Morphine Abstinence

**Figure 1:**
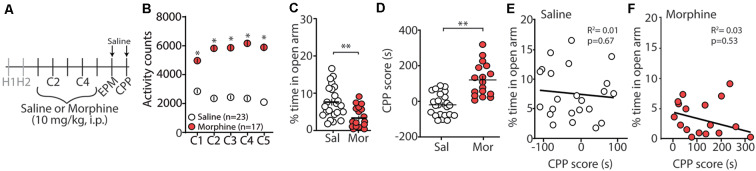
Morphine conditioning in a conditioned place preference (CPP) paradigm elicits anxiety-like behaviors during 24 h abstinence. **(A)** Timeline and drug regimen of the behavioral procedure. Animals underwent 2 days of habituation (H), followed by 5 days of saline or morphine (10 mg/kg, i.p.) conditioning (C), before being subjected to tests measuring anxiety-like behaviors using an elevated plus maze (EPM) 24 h post-conditioning. Twenty-four hours post EPM tests, CPP tests were performed. Animals were injected with saline 30 min before EPM and CPP tests. **(B)** A summary showing that morphine conditioning over 5 days produces robust locomotor sensitization (*F*_(4,152)_ = 17.1, *p* < 0.0001, two-way repeated-measures ANOVA, Bonferroni *post hoc* test). **(C)** A summary showing that morphine (Mor)-conditioned mice spent significantly less time in the open arms of the elevated plus-maze compared to saline (Sal)-conditioned mice 24 h following the last conditioning day (*t*_(38)_ = 3.35, *p* = 0.002, student’s *t*-test). **(D)** A summary showing that morphine conditioning produced reliable CPP (*t*_(38)_ = 5.61, *p* < 0.0001, student’s *t*-test). **(E)** Correlation of the % time in the open arm of the elevated plus-maze and CPP score in saline- or **(F)** morphine-conditioned mice. *p < 0.05, **p < 0.01.

Repeated exposure to morphine increases levels of anxiety both in humans and in animal models of substance use disorders (Gold et al., 1978, 1979; Becker et al., 2017). Additionally, it is posited that relapse to opioids in abstinent patients is caused by negative affective states, thus driving drug-seeking behaviors (Solomon and Corbit, 1978; Koob and Le Moal, 2008; Evans and Cahill, 2016). In an attempt to provide evidence that morphine-induced CPP, using our training paradigm, is mediated, in part, by negative affective states, 24 h following the last morphine conditioning session (Figure 1A), we measured anxiety-like behavior using the elevated plus maze (EPM; Pellow et al., 1985). We found that morphine-treated mice, who showed robust locomotor sensitization by conditioning day 5 (Figure 1B), expressed a significant decrease in the percent time spent in the open arm of the EPM compared to saline-treated controls (*t*_(38)_ = 3.35, *p* = 0.002, student’s *t*-test; Figure 1C). To correlate anxiety levels with CPP scores, mice underwent CPP tests 24 h following EPM tests (Figure 1A). We found that 5 days morphine conditioning elicited significant increases in place preference for the drug-paired compartment (*t*_(38)_ = 5.61, *p* < 0.0001, student’s *t*-test; Figure 1D). However, we found no correlation between anxiety-like behaviors and CPP score in morphine-conditioned mice (Pearson’s correlation coefficient = −0.162; simple linear regression: *F*_(1,15)_ = 0.404, *p* = 0.53, *R*^2^ = 0.03) or in saline-conditioned, control mice (Pearson’s correlation coefficient = −0.095; simple linear regression: *F*_(1,21)_ = 0.191, *p* = 0.67, *R*^2^ = 0.01; Figures 1E,F). Overall, these results suggest that morphine conditioning in a CPP paradigm is sufficient to facilitate anxiety-like behaviors during short-term abstinence, but that the animal’s anxiety-like behavior is not correlated with the amount of time spent in the morphine-paired compartment on CPP test day.

### Ketamine Blocks Morphine-Induced Anxiety-Like Behaviors and Morphine CPP

**Figure 2:**
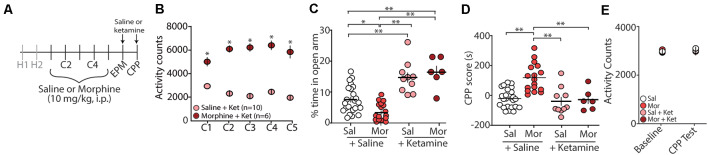
Acute (*R,S*)-ketamine injection produces anxiolytic-like behaviors in mice 24 h after conditioning and blocks morphine-induced CPP. **(A)** Timeline and drug regimen of the behavioral procedure. Saline or (*R,S*)-ketamine (10 mg/kg, i.p.) was injected 30 min before EPM test with the second injection taking place 30 min before the first CPP test. **(B)** A summary showing that morphine conditioning over 5 days (C1–C5) produces robust locomotor sensitization (*F*_(4,56)_ = 12.55, *p* < 0.0001, two-way repeated-measures ANOVA, Bonferroni *post hoc* test). **(C)** A summary showing that (*R,S*)-ketamine significantly increased the time spent in the open arms of the elevated plus-maze in both saline (Sal)- and morphine (Mor)-conditioned mice (*F*_(3,52)_ = 22.2, *p* < 0.0001, one-way ANOVA, Bonferroni *post hoc* test; animals not receiving (*R,S*)-ketamine are the same data as shown in Figure 1C). **(D)** A summary showing that morphine produced reliable CPP at post-conditioning day 2, which was blocked by (*R,S*)-ketamine injected 30 min before testing (*F*_(3,52)_ = 14.04, *p* < 0.0001, one-way ANOVA, Bonferroni *post hoc* test; saline and morphine groups are the same animals as shown in Figure 1D). **(E)** A summary showing the activity counts in the CPP chamber during habituation (baseline) and the CPP test in saline (Sal)- or morphine (Mor)-conditioned mice treated with saline or (*R,S*)-ketamine 30 min before testing (*F*_(3,52)_ = 0.447, *p* = 0.72, two-way repeated-measures ANOVA). *p < 0.05, **p < 0.01.

Evidence suggests that (*R,S*)-ketamine, a noncompetitive NMDA receptor antagonist (Lodge et al., 1982; Kohrs and Durieux, 1998), is an effective treatment for anxiety and substance use disorders (Krupitsky et al., 2002; Ivan Ezquerra-Romano et al., 2018; Taylor et al., 2018). Because of this, we investigated whether an acute injection of (*R,S*)-ketamine (30 min before EPM and CPP testing) would be sufficient to block morphine-induced anxiety-like behaviors and/or morphine-induced CPP (Figure 2A). Following conditioning with morphine, which produced robust locomotor sensitization (Figure 2B), we found that the first (*R,S*)-ketamine injection before the EPM test on post-conditioning day 1 (PC1) significantly increased the percent time in the open arms of the EPM (*F*_(3,52)_ = 22.2, *p* < 0.0001, one-way ANOVA, Bonferroni *post hoc* test; Figure 2C). Additionally, we found that a second (*R,S*)-ketamine injection before CPP tests on post-conditioning day 2 (PC2) was sufficient to prevent morphine-induced CPP (*F*_(3,52)_ = 14.04, *p* < 0.0001, one-way ANOVA, Bonferroni *post hoc* test; Figure 2D), which was likely not attributed to ketamine-induced changes in locomotor activity (*F*_(3,52)_ = 0.447, *p* = 0.72, two-way repeated-measures ANOVA; Figure 2E).

### Acute Ketamine Treatment Blocks the Long-Term Expression of Morphine CPP

**Figure 3:**
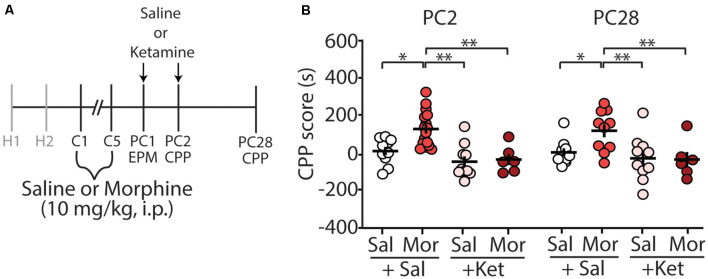
(*R,S*)-ketamine administration during early abstinence is sufficient to prevent the prolonged retention of morphine-induced CPP at post-conditioning day 28. **(A)** Timeline and drug regimen of the behavioral procedure. (*R,S*)-ketamine (10 mg/kg, i.p.) was injected 30 min before the EPM test on post-conditioning day 1 (PC1) and again on the first CPP test on post-conditioning day 2 (PC2; i.e., each mouse received a ketamine injection before the EPM test and a second ketamine injection the next day before the CPP test). The second CPP test was run on PC28. **(B)** A summary showing that morphine produced reliable CPP 28 days post-conditioning, which was blocked by (*R,S*)-ketamine (column factor: *F*_(3,38)_ = 10.25, *p* < 0.0001, two-way repeated-measures ANOVA, Bonferroni *post hoc* test; PC2 data is the same data shown in Figure 2D). Abbreviation: EPM, elevated plus maze; CPP, conditioned place preference. *p < 0.05, **p < 0.01.

We have previously shown that morphine-induced CPP, using the paradigm described in this study, is sufficient to elicit long-lasting CPP for up to 28 days post-conditioning (Graziane et al., 2016). Because of this, we tested whether ketamine administration during early abstinence was sufficient to block the prolonged-expression of morphine-induced CPP (Figure 3A). We found that two injections of (*R,S*)-ketamine, one on post-conditioning day 1 (before elevated arm maze tests) and the second on post-conditioning day 2 (before CPP tests), were sufficient to prevent the prolonged-expression of morphine-induced CPP on PC28 (column factor: *F*_(3,38)_ = 10.25, *p* < 0.0001, two-way repeated-measures ANOVA, Bonferroni *post hoc* test; Figure 3B).

### Acute Ketamine Treatment Prevents the Expression of Sucrose CPP

**Figure 4:**
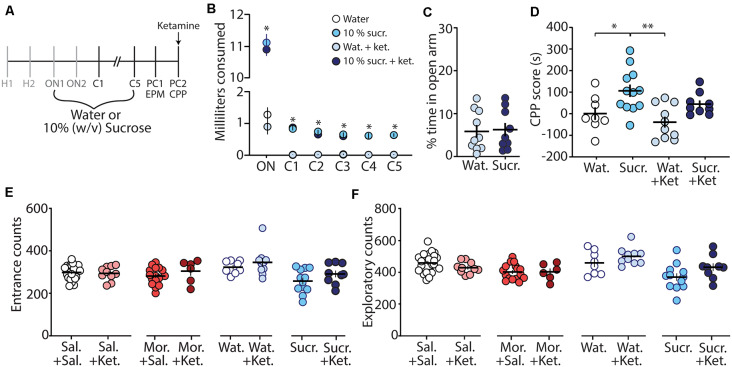
Ketamine administration attenuates sucrose-induced conditioned place preference. **(A)** Timeline and sucrose regimen of the behavioral procedure. Following sucrose oral self-administration in the three-compartment apparatus, mice underwent EPM testing on post-conditioning day 1 (PC1). Twenty-four hours later, mice received no injection or (*R,S*)-ketamine (10 mg/kg, i.p.) 30 min before the conditioned place preference (CPP) test on post-conditioning day 2 (PC2). **(B)** A summary showing the milliliters of water or sucrose consumed for each training session in the least preferred chamber. Groups conditioned with sucrose (i.e., sucrose (sucr.) and sucrose + ketamine (sucr. + ket.) groups) drank significantly more than groups conditioned with water (i.e., water (Wat.) and water+ketamine (Wat. + Ket.) groups; *F*_(15,175)_ = 462.1, *p* < 0.0001, two-way repeated-measures ANOVA, Bonferroni *post hoc* test). **(C)** A summary showing that conditioning with sucrose had no effect on anxiety-like behaviors as both water- and sucrose-conditioned mice displayed similar % time in the open arm of the EPM (*t*_(17)_ = 0.184, *p* = 0.856, student’s *t*-test). **(D)** A summary showing that oral self-administration of sucrose produced CPP at PC2, which was blocked by (*R,S*)-ketamine treatment (*F*_(3,35)_ = 6.31, *p* = 0.0015, one-way ANOVA, Bonferroni *post hoc* test). **(E)** A summary showing that ketamine injections 30 min before the CPP test did not impact entrance counts in the CPP apparatus (Sal. + Sal. vs. Sal. + Ket.: *t*_(31)_ = 0.295, *p* = 0.770; Mor. + Sal. vs. Mor. + Ket.: *t*_(21)_ = 1.13, *p* = 0.272; Wat. + Sal. vs. Wat. + Ket.: *t*_(16)_ = 0.874, *p* = 0.395; Sucr. + Sal. vs. Sucr. + Ket.: *t*_(19)_ = 1.43, *p* = 0.168, student’s *t*-test). **(F)** A summary showing that ketamine injections 30 min before the CPP test did not impact exploratory counts in the CPP apparatus (Sal. + Sal. vs. Sal. + Ket.: *t*_(31)_ = 1.42, *p* = 0.166; Mor. + Sal. vs. Mor. + Ket.: *t*_(21)_ = 0.045, *p* = 0.964; Wat. + Sal. vs. Wat. + Ket.: *t*_(16)_ = 1.26, *p* = 0.226; Sucr. + Sal. vs. Sucr. + Ket.: *t*_(19)_ = 1.80, *p* = 0.088, student’s *t*-test). *p < 0.05, **p < 0.01.

To further investigate whether the ketamine block of morphine CPP is through potential memory impairment and/or anxiolytic effects, we evaluated the effect of ketamine on the CPP of a natural reward (i.e., sucrose). We rationalized that if ketamine blocks morphine CPP by specifically alleviating negative affective states, without impairing memory of drug-context associations, then ketamine would be ineffective at blocking sucrose CPP, a natural reward, which does not evoke anxiety-like behaviors (Figure 4C). To test this, we conditioned mice over 7 days (Figure 4A) to orally self-administer water (controls) or sucrose in the least preferred compartment of the CPP chamber (see “Materials and Methods” section for conditioning paradigm). Mice conditioned with sucrose drank significantly more than mice conditioned with water over all conditioning days (*F*_(15,175)_ = 462.1, *p* < 0.0001, two-way repeated-measures ANOVA, Bonferroni *post hoc* test; Figure 4B). The water consumed in the most preferred chamber during conditioning days 1–5 did not differ between groups (*F*_(12,140)_ = 0.596, *p* = 0.843, two-way repeated-measures ANOVA; Supplementary Figure S1). On post-conditioning day 1 (PC1), anxiety-like behavior was measured using the EPM. We found that the percent time in the open arm of the EPM in sucrose-conditioned mice was not significantly different from mice conditioned with water (*t*_(17)_ = 0.184, *p* = 0.856, student’s *t*-test; Figure 4C) suggesting that sucrose exposure did not elicit anxiety-like behaviors during short-term abstinence. Twenty-four hours later, on post-conditioning day 2 (PC2), water- and sucrose-conditioned mice underwent a CPP test 30 min after receiving an acute injection of (*R,S*)-ketamine (10 mg/kg, i.p.). Our data show that (*R,S*)-ketamine attenuated sucrose-induced CPP on PC2 (*F*_(3,35)_ = 6.31, *p* = 0.0015, one-way ANOVA, Bonferroni *post hoc* test; Figure 4D) and this ketamine-induced attenuation of sucrose CPP persisted to abstinence day 21 (*F*_(3,32)_ = 5.51, *p* = 0.004, one-way ANOVA, Bonferroni *post hoc* test; Supplementary Figure S2).

Lastly, we investigated whether the ketamine block of morphine-induced anxiety-like behavior and morphine-induced CPP was potentially attributed to ketamine-induced behavioral disinhibition, leading the animal to explore more. To do this, we monitored entrance counts and exploratory counts in the CPP chamber on test day. We found that there was no significant difference in the entrance or exploratory counts in the CPP chamber when comparisons were made between saline vs. ketamine injected mice undergoing the same treatment during conditioning (Figures 4E,F). These results suggest that the effects of ketamine on morphine-driven behaviors are unlikely mediated by behavioral disinhibition.

## Discussion

Our results show that the percent time spent in the open arms of the elevated plus-maze is decreased in animals conditioned with morphine. Additionally, we show that acute injection of (*R,S*)-ketamine 30 min before the EPM and CPP tests is sufficient to block morphine-induced anxiety-like behaviors and morphine-induced CPP (post-conditioning day 2 through post-conditioning day 28), as well as attenuates sucrose-induced CPP (post-conditioning day 2 through post-conditioning day 21). We further find that ketamine, at least in the dose tested here, does not alter behavioral disinhibition in either morphine-CPP or sucrose-CPP mice. Together these findings indicate that ketamine may inhibit morphine CPP behaviors, at least in part, *via* reductions in withdrawal-induced anxiety-like behaviors. Our data do not, however, rule out the possibility that ketamine-induced effects on morphine CPP may also be mediated in part by impairing memory of morphine-context associations.

### Anxiety-Like Behaviors During Morphine Abstinence

Morphine possesses anxiolytic-like properties during initial exposure (Kõks et al., 1999; Sasaki et al., 2002; Shin et al., 2003). However, during opioid abstinence, symptoms of anxiety (Gold et al., 1978, 1979; Li et al., 2009; Shi et al., 2009) or anxiety-like behaviors are observed (Cabral et al., 2009; Becker et al., 2017). Here, we show that 24 h following repeated morphine injections (once a day for 5 days), mice display anxiety-like behaviors in the elevated plus-maze (Figure 1C). These results are similar to previous studies showing escalating doses of morphine over 6 days induce anxiety-like behaviors in the marble burying task (Becker et al., 2017). Additionally, our observed morphine-induced anxiety-like behavior is timed with anxiogenic neurobiological responses that occur during acute opioid abstinence including, increases in norepinephrine release in the extended amygdala (Fuentealba et al., 2000; Aston-Jones and Harris, 2004), norepinephrine-induced modulation of the extended amygdala (Aston-Jones et al., 1999; Delfs et al., 2000; Smith and Aston-Jones, 2008), activation of the amygdalar corticotrophin-releasing factor (CRF) system (Heinrichs et al., 1995; Maj et al., 2003), and decreases in dopamine transmission (Diana et al., 1995). However, the observed morphine-induced anxiety-like behavior may be dependent upon morphine exposure as it has been shown that morphine does not elicit anxiety-like behaviors following three morphine injections (10 mg/kg) occurring every other day (Benturquia et al., 2007). This may be related to neurobiological mechanisms associated with different drug exposure regimens. We have previously shown that morphine exposure significantly increases the expression of silent synapses, excitatory glutamatergic synapses that express functional NMDA receptors, but lack functional α-amino-3-hydroxy-5-methyl-4-isoxazolepropionic acid (AMPA) receptors (Hanse et al., 2013), in the nucleus accumbens shell. We found that this increase in silent synapse expression is observed 24 h after the last of five morphine injections (once a day for 5 days), but not 24 h after the last of three morphine injections (once a day for 3 days; Graziane et al., 2016; Hearing et al., 2018; McDevitt and Graziane, 2018). Future experiments will be required to test whether this morphine-induced change in the nucleus accumbens shell regulates morphine-induced anxiety-like behaviors.

The observed anxiety-like behaviors following morphine conditioning in a three-chamber apparatus (Figure 1F) may suggest that animals seek the drug-paired chamber as a consequence of negative reinforcement to alleviate aversive affective states facilitated by opioid abstinence. Importantly, our injection regimen of morphine 10 mg/kg once a day for 5 consecutive days does not induce signs of somatic withdrawal in mice including jumping, wet dog shakes, teeth chattering, rearing, tremor, diarrhea, or mastication (Gallego et al., 2010). This coincides with the lack of observed somatic withdrawal symptoms following a more prolonged injection regimen of five daily morphine (10 mg/kg, i.p.) injections over 4 weeks (Robinson and Kolb, 1999). Although more studies are required, it is plausible that specific opioid dosing regimens may be implemented in a preclinical setting to separate opioid-induced negative affective states (e.g., anxiety) from confounds induced by somatic signs of opioid withdrawal, which are ineffective at reinstating opioid seeking or morphine CPP in opioid-dependent rodents (Shaham et al., 1996; Lu et al., 2005) as well as in humans (Miller et al., 1979). Separating opioid-induced negative affective states (e.g., anxiety) from confounds induced by somatic signs of opioid withdrawal is not a new idea and has been demonstrated previously with doses of naloxone (used to precipitate opioid withdrawal) that were sub-threshold for somatic signs of opioid withdrawal (Gracy et al., 2001).

Based on our results, it would be expected that facilitating a negative affective state during morphine abstinence would enhance the expression of morphine CPP. However, evidence suggests that this is not the case, as forced swim stress, which would be expected to elicit a strong negative affective state, immediately before CPP testing in morphine-conditioned animals has either no effect on morphine CPP (Attarzadeh-Yazdi et al., 2013) or significantly decreases morphine CPP (Haghparast et al., 2014). Additionally, corticosterone administration, which is expected to facilitate depression-like behaviors (Gregus et al., 2005), before CPP tests do not affect morphine CPP (Attarzadeh-Yazdi et al., 2013). These results are surprising especially considering the robust effect of stressful stimuli in reinstating morphine CPP in extinguished rodents (Ribeiro Do Couto et al., 2006; Wang et al., 2006; Karimi et al., 2014). Possibly, morphine CPP tested during abstinence (e.g., Attarzadeh-Yazdi et al., 2013) reaches a ceiling effect, making it unlikely that exposure to a stressor (e.g., forced swim) will enhance the CPP score (i.e., occlusion). It is also possible that the stressor elicits a decreased locomotor state potentially resulting in reduced morphine CPP (e.g., Haghparast et al., 2014).

### Ketamine’s Effects on Anxiety-Like Behaviors

Ketamine has recently been shown to be a potentially effective treatment for anxiety disorders (Glue et al., 2018; Shadli et al., 2018; Taylor et al., 2018). In humans, ketamine displays a biphasic dose-effect on anxiety, with low doses decreasing anxiety and higher doses increasing anxiety (Jansen, 1989; Krystal et al., 1994). Likewise, in rodents, ketamine induces anxiolytic-like behaviors (Engin et al., 2009; Zhang et al., 2015; Fraga et al., 2018) as well as anxiogenic-like phenotypes likely dependent upon the dose, the temporal relationship between ketamine injection and test onset, and rodent species (Silvestre et al., 1997; da Silva et al., 2010). Here, we demonstrate that in C57BL/6J mice, acute injection of ketamine at 10 mg/kg, i.p. Thirty minutes before testing is sufficient to block morphine-induced anxiety-like behaviors during a 24 h abstinence period (Figure 2C). Additionally, we find that ketamine significantly increases the percent time in the open arm of the elevated plus-maze in mice conditioned with saline. This significant change observed in saline conditioned animals suggests that ketamine, at the dose and temporal relationship of ketamine injection and test onset, is sufficient to overcome baseline anxiety-like behaviors in animals exposed to a novel environment (i.e., EPM).

Despite the evidence suggesting that the antagonistic effects of ketamine on NMDA receptors in the bed nucleus of the stria terminalis attenuate negative affective states (Louderback et al., 2013), the mechanisms mediating the observed anxiolytic-like effects are unknown. In addition to acting as a non-competitive antagonist to NMDA receptors in the extended amygdala, evidence suggests that ketamine interacts with hyperpolarization-activated cyclic nucleotide-gated (HCN) channels as well as dopamine, serotonin, sigma, opioid, and cholinergic receptors (Scheller et al., 1996; Cai et al., 1997; Kubota et al., 1999; Lydic and Baghdoyan, 2002; Wang et al., 2012; Zanos et al., 2018). Additionally, ketamine metabolites are biologically active as antagonists to NMDA receptors (Ebert et al., 1997) and α7 nicotinic acetylcholine receptors (Moaddel et al., 2013), while also possessing agonistic activity for AMPA receptors (Zanos et al., 2016; Tyler et al., 2017). Because of the undiscriminating activity of ketamine and its metabolites, it has been difficult to pinpoint how ketamine influences anxiety states both in humans and in preclinical models.

### Ketamine’s Effects on Morphine-Induced Conditioned Place Preference

Using a paradigm known to induce robust CPP for up to 28 days post-conditioning (Graziane et al., 2016), we show that acute injection of ketamine 30 min before the CPP test on abstinence day 2 is sufficient to block morphine-induced CPP. These results are not likely caused by changes in locomotor activity as activity counts during habituation (baseline) were not significantly different from activity counts measured following ketamine administration (Figure 2E). Our results are in line with previous publications demonstrating that ketamine blocks morphine-induced CPP in mice (Suzuki et al., 2000). However, the effects on locomotor activity are conflicting. Whereas, our results and those from previous publications show that ketamine does not influence locomotor activity (Lindholm et al., 2012), others have found that locomotor activity is increased (Filibeck and Castellano, 1980) or decreased following ketamine administration (Akillioglu et al., 2012). These discrepancies are likely due to the temporal relationship between ketamine treatment and test onset. Here, we performed our tests 30 min following ketamine injection similar to previous studies (Lindholm et al., 2012), while tests performed 5 min or 15 min following ketamine administration appear to increase or decrease locomotor activity, respectively (Filibeck and Castellano, 1980; Akillioglu et al., 2012). The half-life of ketamine is ~13–25 min in mice following i.p. administration (Maxwell et al., 2006; Zanos et al., 2016; Ganguly et al., 2018). Therefore, possibly the locomotor effects observed are due to ketamine action before metabolism, while the effects on negative affect are potentially attributed to ketamine metabolites including hydroxynorketamine (Li et al., 2015; Zanos et al., 2016). This hypothesis will need to be tested in future experiments. Moreover, our results are based on using a fixed dose of ketamine at 10 mg/kg, thus preventing dose-response observations. Future investigations are required to test how varying ketamine doses may influence morphine-induced CPP as well as morphine-induced anxiety-like behaviors.

Based on our findings that ketamine elicited anxiolytic-like behaviors following an acute injection, perhaps, the acute administration of ketamine was sufficient to prevent a negative affective state during 24 h morphine abstinence, thus facilitating the lack of motivation to seek a context paired with a drug reward (i.e., morphine-induced CPP). It is also plausible that the block of morphine-induced CPP by ketamine may be mediated by its effects on cognition and memory, thus blocking the recall of morphine-context associations (Ghoneim et al., 1985; Newcomer et al., 1999; Morgan et al., 2004; Malhotra et al., 1996; Pfenninger et al., 2002). Evidence suggests that ketamine-induced deficits in cognitive functioning and memory occur during the consolidation or, as shown in rodents, reconsolidation (Zhai et al., 2008) of information, rather than the retrieval of already learned associations (Honey et al., 2005). Furthermore, it has been shown in rodent models that the memory impairing effects of ketamine are not attributed to its effects on memory retrieval (Goulart et al., 2010). Therefore, acute injection of ketamine before CPP tests is not likely to influence already encoded morphine-context associations. However, we found that ketamine was effective at attenuating sucrose-induced CPP, despite the lack of anxiety-like behavior induced by sucrose conditioning (Figures 4C,D). Therefore, these data suggest that ketamine can interfere with memory associated with Pavlovian learning when administered before retrieval of already learned associations. We acknowledge that our data do not unequivocally demonstrate that the ketamine-induced block of morphine CPP is solely mediated by impairing already learned associations. Therefore, future studies are required to test whether blocking only morphine-induced negative affective states are sufficient to prevent morphine CPP.

Lastly, our data suggest that the effects of ketamine on morphine-induced anxiety-like behavior and morphine CPP are not likely a result of ketamine-induced behavioral disinhibition, which would be expected to increase exploratory behaviors. We found that ketamine did not affect entrance counts or exploratory behaviors in the CPP apparatus (Figures 4E,F).

Overall, our data suggest that ketamine may influence morphine CPP by altering negative affective states as well as by altering the memory of learned associations. However, this does not rule out that ketamine’s effects on morphine-induced CPP may be mediated by other mechanisms of action as ketamine has proven effective for treating pain (Weisman, 1971; Laskowski et al., 2011; Jonkman et al., 2017), depression (Khorramzadeh and Lotfy, 1973; Sofia and Harakal, 1975), and inflammation (Roytblat et al., 1998; Beilin et al., 2007; Loix et al., 2011).

### Ketamine as a Treatment Option for Substance Use Disorders

There is growing clinical and preclinical evidence that ketamine may be a potential treatment option for substance use disorders (Ivan Ezquerra-Romano et al., 2018; Jones et al., 2018). Through the use of Ketamine Assisted Psychotherapy (KAP; Ivan Ezquerra-Romano et al., 2018), alcohol-dependent patients (Krupitsky and Grinenko, 1997; Kolp et al., 2006), heroin-dependent patients (Krupitsky et al., 2002, 2007), and cocaine-dependent patients (Dakwar et al., 2017) showed greater rates of abstinence and reductions in drug craving. These results have been echoed in preclinical models of substance use disorders as acute ketamine injections significantly attenuate alcohol self-administration (Sabino et al., 2013) and prevent the reconsolidation of morphine-induced CPP (Zhai et al., 2008). Here, we discovered a novel and unexpected loss of long-term expression of morphine-induced CPP (PC28) in animals injected with (*R,S*)-ketamine at time points corresponding to 24 and 48 h post CPP conditioning. These results demonstrate the profound effect that (*R,S*)-ketamine has on reward-related behaviors and opens up many avenues including, investigating temporal effects of ketamine treatment at later time points following conditioning, the neurocircuit mechanisms mediating this prolonged ketamine effect on morphine-induced CPP, and the specificity for drug-context associations vs. other forms of memory. With the ever-increasing use of ketamine as an antidepressant in major depressive disorder (Berman et al., 2000; Diazgranados et al., 2010; Ibrahim et al., 2011; Zarate et al., 2012; Murrough et al., 2013b), applying its therapeutic use to patients suffering from substance use disorders holds potential value as an alternative treatment option.

### Limitations to the Use of Ketamine as a Treatment Option for Substance Use Disorders

Despite its therapeutic value, ketamine has undesirable side effects including drowsiness, confusion, dizziness, and dissociative psychiatric side effects (Zarate et al., 2006; Diazgranados et al., 2010; Ibrahim et al., 2011; Murrough et al., 2013a). Additionally, evidence suggests that ketamine impairs cognition and memory (Harris et al., 1975; Ghoneim et al., 1985; Malhotra et al., 1996; Newcomer et al., 1999; Pfenninger et al., 2002; Morgan et al., 2004; Honey et al., 2005; Mathew et al., 2010; Driesen et al., 2013) and may cause urological effects (Middela and Pearce, 2011). A limitation of ketamine use as a treatment option for substance use disorders is its abuse potential (Liu et al., 2016). However, controlled studies in patients addressing the abuse potential of low-dose ketamine are lacking and if the long-lasting ketamine effects shown here in mice translate to human patients, the abuse liability can be mitigated by monthly physician-administered injections.

## Conclusion

Here, we found that morphine conditioning in a three-compartment apparatus that elicits robust CPP was sufficient to evoke anxiety-like behaviors in mice. Additionally, we provided evidence that acute ketamine pretreatment produces anxiolytic-like behaviors and blocks morphine-induced CPP for a prolonged period, suggesting that ketamine is a potential option for attenuating negative reinforcement as well as learned associations that are implicated in substance use disorders.

## Data Availability Statement

The datasets generated for this study are available on request to the corresponding author.

## Ethics Statement

The animal study was reviewed and approved by The Pennsylvania State University College of Medicine Institutional Animal Care and Use Committee.

## Author Contributions

GM, HG, HJ, DM, SS, YS, and NG designed the experiments, performed the analyses, and wrote the manuscript. HJ, GM, HG, DM, and SS performed behavioral training and testing.

## Conflict of Interest

The authors declare that the research was conducted in the absence of any commercial or financial relationships that could be construed as a potential conflict of interest.

## Supplementary Material

FIGURE S1 A summary showing that there is no significant difference in the amount of water consumed in the most preferred side among all groups (*F*_(12,140)_ = 0.596, *p* = 0.843, two-way repeated-measures ANOVA).

FIGURE S2 (*R,S*)-ketamine administration during early abstinence blocks the prolonged retention of sucrose-induced CPP at post conditioning day 21. **(A)** Time line and drug regimen of the behavioral procedure. (*R,S*)-ketamine (10 mg/kg, i.p.) was injected 30 min prior to the first CPP test on post conditioning day 2 (PC2). **(B)** Summary showing that oral self-administration of sucrose produced CPP for the sucrose-paired context 21 days after conditioning. This prolonged expression of sucrose-induced CPP was blocked by (*R,S*)-ketamine when injected 30 min prior to testing on PC2 (*F*_(3,32)_ = 5.51, *p* = 0.004, one-way ANOVA, Bonferroni *post hoc* test). **p* < 0.05, ***p* < 0.01.

## References

[B1] Akillioglu K. Melik E. B. Melik E. Boga A. (2012). Effect of ketamine on exploratory behaviour in BALB/C and C57BL/6 mice. Pharmacol. Biochem. Behav. 100, 513–517. 10.1016/j.pbb.2011.10.014 22037409

[B2] Aston-Jones G. Harris G. C. (2004). Brain substrates for increased drug seeking during protracted withdrawal. Neuropharmacology 47, 167–179. 10.1016/j.neuropharm.2004.06.020 15464135

[B3] Aston-Jones G. Delfs J. M. Druhan J. Zhu Y. (1999). The bed nucleus of the stria terminalis. A target site for noradrenergic actions in opiate withdrawal. Ann. N Y Acad. Sci. 877, 486–498. 10.1111/j.1749-6632.1999.tb09284.x 10415666

[B4] Attarzadeh-Yazdi G. Karimi S. Azizi P. Yazdi-Ravandi S. Hesam S. Haghparast A. (2013). Inhibitory effects of forced swim stress and corticosterone on the acquisition but not expression of morphine-induced conditioned place preference: involvement of glucocorticoid receptor in the basolateral amygdala. Behav. Brain Res. 252, 339–346. 10.1016/j.bbr.2013.06.018 23800381

[B5] Baker T. B. Piper M. E. McCarthy D. E. Majeskie M. R. Fiore M. C. (2004). Addiction motivation reformulated: an affective processing model of negative reinforcement. Psychol. Rev. 111, 33–51. 10.1037/0033-295x.111.1.33 14756584

[B6] Becker J. A. J. Kieffer B. L. Le Merrer J. (2017). Differential behavioral and molecular alterations upon protracted abstinence from cocaine versus morphine, nicotine, THC and alcohol. Addict. Biol. 22, 1205–1217. 10.1111/adb.12405 27126842 PMC5085894

[B7] Beilin B. Rusabrov Y. Shapira Y. Roytblat L. Greemberg L. Yardeni I. Z.. (2007). Low-dose ketamine affects immune responses in humans during the early postoperative period. Br. J. Anaesth. 99, 522–527. 10.1093/bja/aem218 17681970

[B8] Benturquia N. Le Guen S. Canestrelli C. Lagente V. Apiou G. Roques B. P.. (2007). Specific blockade of morphine- and cocaine-induced reinforcing effects in conditioned place preference by nitrous oxide in mice. Neuroscience 149, 477–486. 10.1016/j.neuroscience.2007.08.003 17905521

[B9] Berman R. M. Cappiello A. Anand A. Oren D. A. Heninger G. R. Charney D. S.. (2000). Antidepressant effects of ketamine in depressed patients. Biol. Psychiatry 47, 351–354. 10.1016/s0006-3223(99)00230-9 10686270

[B10] Bilbey D. L. Salem H. Grossman M. H. (1960). The anatomical basis of the straub phenomenon. Br. J. Pharmacol. Chemother. 15, 540–543. 10.1111/j.1476-5381.1960.tb00277.x 19108140 PMC1482265

[B11] Bohn L. M. Gainetdinov R. R. Sotnikova T. D. Medvedev I. O. Lefkowitz R. J. Dykstra L. A.. (2003). Enhanced rewarding properties of morphine, but not cocaine, in β(arrestin)-2 knock-out mice. J. Neurosci. 23, 10265–10273. 10.1523/JNEUROSCI.23-32-10265.2003 14614085 PMC6741024

[B12] Cabral A. Ruggiero R. N. Nobre M. J. Brañdao M. L. Castilho V. M. (2009). GABA and opioid mechanisms of the central amygdala underlie the withdrawal-potentiated startle from acute morphine. Prog. Neuropsychopharmacol. Biol. Psychiatry 33, 334–344. 10.1016/j.pnpbp.2008.12.012 19150477

[B13] Cai Y.-C. Ma L. Fan G.-H. Zhao J. Jiang L.-Z. Pei G. (1997). Activation of N-methyl-d-aspartate receptor attenuates acute responsiveness of δ-opioid receptors. Mol. Pharmacol. 51, 583–587. 10.1124/mol.51.4.583 9106622

[B14] Conklin C. A. Perkins K. A. (2005). Subjective and reinforcing effects of smoking during negative mood induction. J. Abnorm. Psychol. 114, 153–164. 10.1037/0021-843x.114.1.153 15709822

[B15] Cooney N. L. Litt M. D. Morse P. A. Bauer L. O. Gaupp L. (1997). Alcohol cue reactivity, negative-mood reactivity and relapse in treated alcoholic men. J. Abnorm. Psychol. 106, 243–250. 10.1037/0021-843x.106.2.243 9131844

[B16] da Silva F. C. C. do Carmo De Oliveira Cito M. da Silva M. I. G. Moura B. A. De Aquino Neto M. R. Feitosa M. L.. (2010). Behavioral alterations and pro-oxidant effect of a single ketamine administration to mice. Brain Res. Bull. 83, 9–15. 10.1016/j.brainresbull.2010.05.011 20600677

[B17] Dakwar E. Hart C. L. Levin F. R. Nunes E. V. Foltin R. W. (2017). Cocaine self-administration disrupted by the N-methyl-D-aspartate receptor antagonist ketamine: a randomized, crossover trial. Mol. Psychiatry 22, 76–81. 10.1038/mp.2016.39 27090301 PMC5435123

[B18] Dawson G. R. Tricklebank M. D. (1995). Use of the elevated plus maze in the search for novel anxiolytic agents. Trends Pharmacol. Sci. 16, 33–36. 10.1016/s0165-6147(00)88973-7 7762079

[B19] Delfs J. M. Zhu Y. Druhan J. P. Aston-Jones G. (2000). Noradrenaline in the ventral forebrain is critical for opiate withdrawal-induced aversion. Nature 403, 430–434. 10.1038/35000212 10667795

[B20] Diana M. Pistis M. Muntoni A. Gessa G. (1995). Profound decrease of mesolimbic dopaminergic neuronal activity in morphine withdrawn rats. J. Pharmacol. Exp. Ther. 272, 781–785. 10.1097/00008877-199505001-00100 7853194

[B21] Diazgranados N. Ibrahim L. Brutsche N. E. Newberg A. Kronstein P. Khalife S.. (2010). A randomized add-on trial of an N-methyl-D-aspartate antagonist in treatment-resistant bipolar depression. Arch. Gen. Psychiatry 67, 793–802. 10.1001/archgenpsychiatry.2010.90 20679587 PMC3000408

[B22] Driesen N. R. McCarthy G. Bhagwagar Z. Bloch M. H. Calhoun V. D. D’Souza D. C.. (2013). The impact of NMDA receptor blockade on human working memory-related prefrontal function and connectivity. Neuropsychopharmacology 38, 2613–2622. 10.1038/npp.2013.170 23856634 PMC3828532

[B23] Ebert B. Mikkelsen S. Thorkildsen C. Borgbjerg F. M. (1997). Norketamine, the main metabolite of ketamine, is a non-competitive NMDA receptor antagonist in the rat cortex and spinal cord. Eur. J. Pharmacol. 333, 99–104. 10.1016/s0014-2999(97)01116-3 9311667

[B24] Engin E. Treit D. Dickson C. T. (2009). Anxiolytic- and antidepressant-like properties of ketamine in behavioral and neurophysiological animal models. Neuroscience 161, 359–369. 10.1016/j.neuroscience.2009.03.038 19321151

[B26] Evans C. J. Cahill C. M. (2016). Neurobiology of opioid dependence in creating addiction vulnerability. F1000Res. 5:1748. 10.12688/f1000research.8369.1 27508068 PMC4955026

[B27] Filibeck U. Castellano C. (1980). Strain dependent effects of ketamine on locomotor activity and antinociception in mice. Pharmacol. Biochem. Behav. 13, 443–447. 10.1016/0091-3057(80)90252-x 7422699

[B28] Fox H. C. Bergquist K. L. Hong K. I. Sinha R. (2007). Stress-induced and alcohol cue-induced craving in recently abstinent alcohol-dependent individuals. Alcohol. Clin. Exp. Res. 31, 395–403. 10.1111/j.1530-0277.2006.00320.x 17295723

[B29] Fraga D. B. Olescowicz G. Moretti M. Siteneski A. Tavares M. K. Azevedo D.. (2018). Anxiolytic effects of ascorbic acid and ketamine in mice. J. Psychiatr. Res. 100, 16–23. 10.1016/j.jpsychires.2018.02.006 29475017

[B30] Freet C. S. Wheeler R. A. Leuenberger E. Mosblech N. A. Grigson P. S. (2013). Fischer rats are more sensitive than Lewis rats to the suppressive effects of morphine and the aversive κ-opioid agonist spiradoline. Behav. Neurosci. 127, 763–770. 10.1037/a0033943 24128363 PMC3973147

[B31] Fuentealba J. A. Forray M. I. Gysling K. (2000). Chronic morphine treatment and withdrawal increase extracellular levels of norepinephrine in the rat bed nucleus of the stria terminalis. J. Neurochem. 75, 741–748. 10.1046/j.1471-4159.2000.0750741.x 10899950

[B32] Gallego X. Murtra P. Zamalloa T. Canals J. M. Pineda J. Amador-Arjona A.. (2010). Increased opioid dependence in a mouse model of panic disorder. Front. Behav. Neurosci. 3:60. 10.3389/neuro.08.060.2009 20204153 PMC2831706

[B33] Ganguly S. Panetta J. C. Roberts J. K. Schuetz E. G. (2018). Ketamine pharmacokinetics and pharmacodynamics are altered by P-glycoprotein and breast cancer resistance protein efflux transporters in mice. Drug Metab. Dispos. 46, 1014–1022. 10.1124/dmd.117.078360 29674491 PMC5992966

[B34] Ghoneim M. M. Hinrichs J. V. Mewaldt S. P. Petersen R. C. (1985). Ketamine: behavioral effects of subanesthetic doses. J. Clin. Psychopharmacol. 5, 70–77. 10.1097/00004714-198504000-00003 3988972

[B35] Glue P. Neehoff S. M. Medlicott N. J. Gray A. Kibby G. McNaughton N. (2018). Safety and efficacy of maintenance ketamine treatment in patients with treatment-refractory generalised anxiety and social anxiety disorders. J. Psychopharmacol. 32, 663–667. 10.1177/0269881118762073 29561204

[B36] Gold M. S. Redmond D. E. Jr. Kleber H. D. (1978). Clonidine blocks acute opiate-withdrawal symptoms. Lancet 2, 599–602. 10.1016/s0140-6736(78)92823-4 80526

[B37] Gold M. S. Redmond D. E. Jr. Kleber H. D. (1979). Noradrenergic hyperactivity in opiate withdrawal supported by clonidine reversal of opiate withdrawal. Am. J. Psychiatry 136, 100–102. 10.1176/ajp.136.1.100 364997

[B38] Goulart B. K. de Lima M. N. M. de Farias C. B. Reolon G. K. Almeida V. R. Quevedo J.. (2010). Ketamine impairs recognition memory consolidation and prevents learning-induced increase in hippocampal brain-derived neurotrophic factor levels. Neuroscience 167, 969–973. 10.1016/j.neuroscience.2010.03.032 20338225

[B39] Gracy K. N. Dankiewicz L. A. Koob G. F. (2001). Opiate withdrawal-induced fos immunoreactivity in the rat extended amygdala parallels the development of conditioned place aversion. Neuropsychopharmacology 24, 152–160. 10.1016/s0893-133x(00)00186-x 11120397

[B40] Graziane N. M. Sun S. Wright W. J. Jang D. Liu Z. Huang Y. H.. (2016). Opposing mechanisms mediate morphine- and cocaine-induced generation of silent synapses. Nat. Neurosci. 19, 915–925. 10.1038/nn.4313 27239940 PMC4925174

[B41] Gregus A. Wintink A. J. Davis A. C. Kalynchuk L. E. (2005). Effect of repeated corticosterone injections and restraint stress on anxiety and depression-like behavior in male rats. Behav. Brain Res. 156, 105–114. 10.1016/j.bbr.2004.05.013 15474655

[B42] Gremel C. M. Gabriel K. I. Cunningham C. L. (2006). Topiramate does not affect the acquisition or expression of ethanol conditioned place preference in DBA/2J or C57BL/6J mice. Alcohol. Clin. Exp. Res. 30, 783–790. 10.1111/j.1530-0277.2006.00091.x 16634846

[B43] Grisel J. E. Bartels J. L. Allen S. A. Turgeon V. L. (2008). Influence of β-endorphin on anxious behavior in mice: interaction with EtOH. Psychopharmacology 200, 105–115. 10.1007/s00213-008-1161-4 18604523 PMC2818628

[B44] Haghparast A. Fatahi Z. Alamdary S. Z. Reisi Z. Khodagholi F. (2014). Changes in the levels of p-ERK, p-CREB, and c-fos in rat mesocorticolimbic dopaminergic system after morphine-induced conditioned place preference: the role of acute and subchronic stress. Cell. Mol. Neurobiol. 34, 277–288. 10.1007/s10571-013-0011-z 24292370 PMC11488949

[B45] Handley S. L. McBlane J. W. (1993). An assessment of the elevated X-maze for studying anxiety and anxiety-modulating drugs. J. Pharmacol. Toxicol. Methods 29, 129–138. 10.1016/1056-8719(93)90063-k 8103377

[B46] Hanse E. Seth H. Riebe I. (2013). AMPA-silent synapses in brain development and pathology. Nat. Rev. Neurosci. 14, 839–850. 10.1038/nrn3642 24201185

[B47] Harris J. A. Biersner R. J. Edwards D. Bailey L. W. (1975). Attention, learning, and personality during ketamine emergence: a pilot study. Anesth. Analg. 54, 169–172. 10.1213/00000539-197503000-00001 1092205

[B48] Hearing M. Graziane N. Dong Y. Thomas M. J. (2018). Opioid and psychostimulant plasticity: targeting overlap in nucleus accumbens glutamate signaling. Trends Pharmacol. Sci. 39, 276–294. 10.1016/j.tips.2017.12.004 29338873 PMC5818297

[B49] Heinrichs S. C. Menzaghi F. Schulteis G. Koob G. F. Stinus L. (1995). Suppression of corticotropin-releasing factor in the amygdala attenuates aversive consequences of morphine withdrawal. Behav. Pharmacol. 6, 74–80. 10.1097/00008877-199501000-00011 11224314

[B50] Honey G. D. Honey R. A. Sharar S. R. Turner D. C. Pomarol-Clotet E. Kumaran D.. (2005). Impairment of specific episodic memory processes by sub-psychotic doses of ketamine: the effects of levels of processing at encoding and of the subsequent retrieval task. Psychopharmacology 181, 445–457. 10.1007/s00213-005-0001-z 15983801

[B51] Huston J. P. Silva M. A. Topic B. Müller C. P. (2013). What’s conditioned in conditioned place preference? Trends Pharmacol. Sci. 34, 162–166. 10.1016/j.tips.2013.01.004 23384389

[B52] Huys Q. J. M. Tobler P. N. Hasler G. Flagel S. B. (2014). “Chapter 3—the role of learning-related dopamine signals in addiction vulnerability,” in Progress in Brain Research, eds Diana M. Di Chiara G. Spano P. (Elsevier), 31–77. 10.1016/B978-0-444-63425-2.00003-9 24968776

[B53] Ibrahim L. Diazgranados N. Luckenbaugh D. A. Machado-Vieira R. Baumann J. Mallinger A. G.. (2011). Rapid decrease in depressive symptoms with an N-methyl-d-aspartate antagonist in ECT-resistant major depression. Prog. Neuropsychopharmacol. Biol. Psychiatry 35, 1155–1159. 10.1016/j.pnpbp.2011.03.019 21466832 PMC3100439

[B54] Ivan Ezquerra-Romano I. Lawn W. Krupitsky E. Morgan C. J. A. (2018). Ketamine for the treatment of addiction: evidence and potential mechanisms. Neuropharmacology 142, 72–82. 10.1016/j.neuropharm.2018.01.017 29339294

[B55] Jansen K. (1989). Near death experience and the NMDA receptor. BMJ 298, 1708–1708. 10.1136/bmj.298.6689.1708-b 2547469 PMC1836765

[B56] Jones J. L. Mateus C. F. Malcolm R. J. Brady K. T. Back S. E. (2018). Efficacy of ketamine in the treatment of substance use disorders: a systematic review. Front. Psychiatry 9, 277–277. 10.3389/fpsyt.2018.00277 30140240 PMC6094990

[B57] Jonkman K. Dahan A. van de Donk T. Aarts L. Niesters M. Van Velzen M. (2017). Ketamine for pain. F1000Res. 6:F1000. 10.12688/f1000research.11372.1 28979762 PMC5609085

[B58] Karimi S. Attarzadeh-Yazdi G. Yazdi-Ravandi S. Hesam S. Azizi P. Razavi Y.. (2014). Forced swim stress but not exogenous corticosterone could induce the reinstatement of extinguished morphine conditioned place preference in rats: involvement of glucocorticoid receptors in the basolateral amygdala. Behav. Brain Res. 264, 43–50. 10.1016/j.bbr.2014.01.045 24508237

[B59] Khorramzadeh E. Lotfy A. O. (1973). The use of ketamine in psychiatry. Psychosomatics 14, 344–346. 10.1016/s0033-3182(73)71306-2 4800188

[B60] Kohrs R. Durieux M. E. (1998). Ketamine: teaching an old drug new tricks. Anesth. Analg. 87, 1186–1193. 10.1097/00000539-199811000-00039 9806706

[B61] Kõks S. Soosaar A. Võikar V. Bourin M. Vasar E. (1999). BOC-CCK-4, CCK(B)receptor agonist, antagonizes anxiolytic-like action of morphine in elevated plus-maze. Neuropeptides 33, 63–69. 10.1054/npep.1999.0015 10657473

[B62] Kolp E. Friedman H. L. Young M. S. Krupitsky E. (2006). Ketamine enhanced psychotherapy: preliminary clinical observations on its effectiveness in treating alcoholism. Hum. Psychol. 34, 399–422. 10.1207/s15473333thp3404_7

[B63] Koo J. W. Lobo M. K. Chaudhury D. Labonté B. Friedman A. Heller E.. (2014). Loss of BDNF signaling in D1R-expressing NAc neurons enhances morphine reward by reducing GABA inhibition. Neuropsychopharmacology 39, 2646–2653. 10.1038/npp.2014.118 24853771 PMC4207344

[B64] Koob G. F. Le Moal M. (2008). Review. Neurobiological mechanisms for opponent motivational processes in addiction. Philos. Trans. R. Soc. Lond. B Biol. Sci. 363, 3113–3123. 10.1098/rstb.2008.0094 18653439 PMC2607326

[B68] Krupitsky E. M. Burakov A. M. Dunaevsky I. V. Romanova T. N. Slavina T. Y. Grinenko A. Y. (2007). Single versus repeated sessions of ketamine-assisted psychotherapy for people with heroin dependence. J. Psychoactive Drugs 39, 13–19. 10.1080/02791072.2007.10399860 17523581

[B65] Krupitsky E. Burakov A. Romanova T. Dunaevsky I. Strassman R. Grinenko A. (2002). Ketamine psychotherapy for heroin addiction: immediate effects and two-year follow-up. J. Subst. Abuse Treat. 23, 273–283. 10.1016/s0740-5472(02)00275-1 12495789

[B67] Krupitsky E. M. Grinenko A. Y. (1997). Ketamine psychedelic therapy (KPT): a review of the results of ten years of research. J. Psychoactive Drugs 29, 165–183. 10.1080/02791072.1997.10400185 9250944

[B69] Krystal J. H. Karper L. P. Seibyl J. P. Freeman G. K. Delaney R. Bremner J. D.. (1994). Subanesthetic effects of the noncompetitive NMDA antagonist, ketamine, in humans. Psychotomimetic, perceptual, cognitive, and neuroendocrine responses. Arch. Gen. Psychiatry 51, 199–214. 10.1001/archpsyc.1994.03950030035004 8122957

[B70] Kubota T. Hirota K. Yoshida H. Takahashi S. Anzawa N. Ohkawa H.. (1999). Effects of sedatives on noradrenaline release from the medial prefrontal cortex in rats. Psychopharmacology 146, 335–338. 10.1007/s002130051125 10541735

[B71] Laskowski K. Stirling A. McKay W. P. Lim H. J. (2011). A systematic review of intravenous ketamine for postoperative analgesia. Can. J. Anaesth. 58, 911–923. 10.1007/s12630-011-9560-0 21773855

[B73] Li X. Martinez-Lozano Sinues P. Dallmann R. Bregy L. Hollmen M. Proulx S.. (2015). Drug pharmacokinetics determined by real-time analysis of mouse breath. Angew. Chem. Int. Ed. Engl. 54, 7815–7818. 10.1002/anie.201503312 26015026

[B72] Li S. X. Shi J. Epstein D. H. Wang X. Zhang X. L. Bao Y. P.. (2009). Circadian alteration in neurobiology during 30 days of abstinence in heroin users. Biol. Psychiatry 65, 905–912. 10.1016/j.biopsych.2008.11.025 19135652

[B74] Lindholm J. S. O. Autio H. Vesa L. Antila H. Lindemann L. Hoener M. C.. (2012). The antidepressant-like effects of glutamatergic drugs ketamine and AMPA receptor potentiator LY 451646 are preserved in BDNF+/− heterozygous null mice. Neuropharmacology 62, 391–397. 10.1016/j.neuropharm.2011.08.015 21867718

[B75] Liu Y. Lin D. Wu B. Zhou W. (2016). Ketamine abuse potential and use disorder. Brain Res. Bull. 126, 68–73. 10.1016/j.brainresbull.2016.05.016 27261367

[B76] Lodge D. Anis N. A. Burton N. R. (1982). Effects of optical isomers of ketamine on excitation of cat and rat spinal neurones by amino acids and acetylcholine. Neurosci. Lett. 29, 281–286. 10.1016/0304-3940(82)90330-5 7048142

[B77] Loix S. De Kock M. Henin P. (2011). The anti-inflammatory effects of ketamine: state of the art. Acta Anaesthesiol. Belg. 62, 47–58. 21612145

[B78] Louderback K. M. Wills T. A. Muglia L. J. Winder D. G. (2013). Knockdown of BNST GluN2B-containing NMDA receptors mimics the actions of ketamine on novelty-induced hypophagia. Transl. Psychiatry 3:e331. 10.1038/tp.2013.103 24301649 PMC4030322

[B79] Lu L. Chen H. Su W. Ge X. Yue W. Su F.. (2005). Role of withdrawal in reinstatement of morphine-conditioned place preference. Psychopharmacology 181, 90–100. 10.1007/s00213-005-2207-5 15739075

[B80] Lydic R. Baghdoyan H. A. (2002). Ketamine and MK-801 decrease acetylcholine release in the pontine reticular formation, slow breathing, and disrupt sleep. Sleep 25, 617–622. 10.1093/sleep/25.6.615 12224840

[B81] Maj M. Turchan J. Śmiałowska M. Przewłocka B. (2003). Morphine and cocaine influence on CRF biosynthesis in the rat central nucleus of amygdala. Neuropeptides 37, 105–110. 10.1016/s0143-4179(03)00021-0 12747942

[B82] Malhotra A. K. Pinals D. A. Weingartner H. Sirocco K. Missar C. D. Pickar D.. (1996). NMDA receptor function and human cognition: the effects of ketamine in healthy volunteers. Neuropsychopharmacology 14, 301–307. 10.1016/0893-133x(95)00137-3 8703299

[B83] Martins S. S. Fenton M. C. Keyes K. M. Blanco C. Zhu H. Storr C. L. (2012). Mood and anxiety disorders and their association with non-medical prescription opioid use and prescription opioid-use disorder: longitudinal evidence from the National Epidemiologic Study on Alcohol and Related Conditions. Psychol. Med. 42, 1261–1272. 10.1017/s0033291711002145 21999943 PMC3513363

[B84] Mathew S. J. Murrough J. W. aan het Rot M. Collins K. A. Reich D. L. Charney D. S. (2010). Riluzole for relapse prevention following intravenous ketamine in treatment-resistant depression: a pilot randomized, placebo-controlled continuation trial. Int. J. Neuropsychopharmacol. 13, 71–82. 10.1017/s1461145709000169 19288975 PMC3883127

[B85] Maxwell C. R. Ehrlichman R. S. Liang Y. Trief D. Kanes S. J. Karp J.. (2006). Ketamine produces lasting disruptions in encoding of sensory stimuli. J. Pharmacol. Exp. Ther. 316, 315–324. 10.1124/jpet.105.091199 16192313

[B86] McDevitt D. S. Graziane N. M. (2018). Neuronal mechanisms mediating pathological reward-related behaviors: A focus on silent synapses in the nucleus accumbens. Pharmacol. Res. 136, 90–96. 10.1016/j.phrs.2018.08.025 30171902

[B87] McDevitt D. S. Graziane N. M. (2019). Timing of morphine administration differentially alters paraventricular thalamic neuron activity. eNeuro 6:ENEURO.0377–0319.2019. 10.1523/eneuro.0377-19.2019 31801741 PMC6920517

[B88] Middela S. Pearce I. (2011). Ketamine-induced vesicopathy: a literature review. Int. J. Clin. Pract. 65, 27–30. 10.1111/j.1742-1241.2010.02502.x 21155941

[B89] Miller D. B. Dougherty J. A. Wikler A. (1979). Interoceptive conditioning through repeated suppression of morphine-abstinence. II. Relapse-testing. Pavlov. J. Biol. Sci. 14, 170–176. 10.1007/BF03001978 45498

[B90] Moaddel R. Abdrakhmanova G. Kozak J. Jozwiak K. Toll L. Jimenez L.. (2013). Sub-anesthetic concentrations of (R,S)-ketamine metabolites inhibit acetylcholine-evoked currents in α7 nicotinic acetylcholine receptors. Eur. J. Pharmacol. 698, 228–234. 10.1016/j.ejphar.2012.11.023 23183107 PMC3534778

[B91] Morgan C. J. Mofeez A. Brandner B. Bromley L. Curran H. V. (2004). Acute effects of ketamine on memory systems and psychotic symptoms in healthy volunteers. Neuropsychopharmacology 29, 208–218. 10.1038/sj.npp.1300342 14603267

[B92] Murrough J. W. Iosifescu D. V. Chang L. C. Al Jurdi R. K. Green C. E. Perez A. M.. (2013a). Antidepressant efficacy of ketamine in treatment-resistant major depression: a two-site randomized controlled trial. Am. J. Psychiatry 170, 1134–1142. 10.1176/appi.ajp.2013.13030392 23982301 PMC3992936

[B93] Murrough J. W. Perez A. M. Pillemer S. Stern J. Parides M. K. aan het Rot M.. (2013b). Rapid and longer-term antidepressant effects of repeated ketamine infusions in treatment-resistant major depression. Biol. Psychiatry 74, 250–256. 10.1016/j.biopsych.2012.06.022 22840761 PMC3725185

[B94] Newcomb M. D. Bentler P. M. (1988). Impact of adolescent drug use and social support on problems of young adults: a longitudinal study. J. Abnorm. Psychol. 97, 64–75. 10.1037//0021-843x.97.1.64 3351114

[B95] Newcomer J. W. Farber N. B. Jevtovic-Todorovic V. Selke G. Melson A. K. Hershey T.. (1999). Ketamine-induced NMDA receptor hypofunction as a model of memory impairment and psychosis. Neuropsychopharmacology 20, 106–118. 10.1016/s0893-133x(98)00067-0 9885791

[B97] O’Brien C. P. (1975). Experimental analysis of conditioning factors in human narcotic addiction. Pharmacol. Rev. 27, 533–543. 1223916

[B98] O’Brien C. P. Childress A. R. McLellan A. T. Ehrman R. (1992). Classical conditioning in drug-dependent humans. Ann. N Y Acad. Sci. 654, 400–415. 10.1111/j.1749-6632.1992.tb25984.x 1632593

[B96] O’Brien E. R. Ternes J. W. (1986). “Classical conditioning in human opioid dependence,” in Behavioral Analysis of Drug Dependence, ed. Goldberg S. I. (Orlando, FL: Academic), 329–356.

[B99] Pellow S. Chopin P. File S. E. Briley M. (1985). Validation of open:closed arm entries in an elevated plus-maze as a measure of anxiety in the rat. J. Neurosci. Methods 14, 149–167. 10.1016/0165-0270(85)90031-7 2864480

[B101] Perkins K. A. Grobe J. E. (1992). Increased desire to smoke during acute stress. Br. J. Addict. 87, 1037–1040. 10.1111/j.1360-0443.1992.tb03121.x 1643396

[B102] Pfenninger E. G. Durieux M. E. Himmelseher S. (2002). Cognitive impairment after small-dose ketamine isomers in comparison to equianalgesic racemic ketamine in human volunteers. Anesthesiology 96, 357–366. 10.1097/00000542-200202000-00022 11818769

[B103] Ribeiro Do Couto B. Aguilar M. A. Manzanedo C. Rodríguez-Arias M. Armario A. Miñarro J. (2006). Social stress is as effective as physical stress in reinstating morphine-induced place preference in mice. Psychopharmacology 185, 459–470. 10.1007/s00213-006-0345-z 16555060

[B104] Robinson T. E. Kolb B. (1999). Morphine alters the structure of neurons in the nucleus accumbens and neocortex of rats. Synapse 33, 160–162. 10.1002/(sici)1098-2396(199908)33:2<160::aid-syn6>3.0.co;2-s 10400894

[B105] Roytblat L. Talmor D. Rachinsky M. Greemberg L. Pekar A. Appelbaum A.. (1998). Ketamine attenuates the interleukin-6 response after cardiopulmonary bypass. Anesth. Analg. 87, 266–271. 10.1097/00000539-199808000-00006 9706914

[B106] Sabino V. Narayan A. R. Zeric T. Steardo L. Cottone P. (2013). mTOR activation is required for the anti-alcohol effect of ketamine, but not memantine, in alcohol-preferring rats. Behav. Brain Res. 247, 9–16. 10.1016/j.bbr.2013.02.030 23466691 PMC3646912

[B107] Sasaki K. Fan L. W. Tien L. T. Ma T. Loh H. H. Ho I. K. (2002). The interaction of morphine and γ-aminobutyric acid (GABA)ergic systems in anxiolytic behavior: using mu-opioid receptor knockout mice. Brain Res. Bull. 57, 689–694. 10.1016/s0361-9230(01)00785-7 11927374

[B108] Scheller M. Bufler J. Hertle I. Schneck H. J. Franke C. Kochs E. (1996). Ketamine blocks currents through mammalian nicotinic acetylcholine receptor channels by interaction with both the open and the closed state. Anesth. Analg. 83, 830–836. 10.1097/00000539-199610000-00031 8831330

[B109] Shadli S. M. Kawe T. Martin D. Mcnaughton N. Neehoff S. Glue P. (2018). Ketamine effects on EEG during therapy of treatment-resistant generalized anxiety and social anxiety. Int. J. Neuropsychopharmacol. 21, 717–724. 10.1093/ijnp/pyy032 29718262 PMC6070106

[B110] Shaham Y. Rajabi H. Stewart J. (1996). Relapse to heroin-seeking in rats under opioid maintenance: the effects of stress, heroin priming, and withdrawal. J. Neurosci. 16, 1957–1963. 10.1523/jneurosci.16-05-01957.1996 8774462 PMC6578687

[B111] Shi J. Li S. X. Zhang X. L. Wang X. Le Foll B. Zhang X. Y.. (2009). Time-dependent neuroendocrine alterations and drug craving during the first month of abstinence in heroin addicts. Am. J. Drug Alcohol Abuse 35, 267–272. 10.1080/00952990902933878 19591065

[B112] Shin I. C. Kim H. C. Swanson J. Hong J. T. Oh K. W. (2003). Anxiolytic effects of acute morphine can be modulated by nitric oxide systems. Pharmacology 68, 183–189. 10.1159/000070457 12837972

[B113] Silvestre J. S. Nadal R. Pallares M. Ferre N. (1997). Acute effects of ketamine in the holeboard, the elevated-plus maze and the social interaction test in Wistar rats. Depress Anxiety 5, 29–33. 10.1002/(sici)1520-6394(1997)5:1<29::aid-da5>3.0.co;2-0 9250438

[B114] Sinha R. (2008). Chronic stress, drug use, and vulnerability to addiction. Ann. N Y Acad. Sci. 1141, 105–130. 10.1196/annals.1441.030 18991954 PMC2732004

[B115] Smith R. J. Aston-Jones G. (2008). Noradrenergic transmission in the extended amygdala: role in increased drug-seeking and relapse during protracted drug abstinence. Brain Struct. Funct. 213, 43–61. 10.1007/s00429-008-0191-3 18651175 PMC3632504

[B116] Sofia R. D. Harakal J. J. (1975). Evaluation of ketamine HCl for anti-depressant activity. Arch. Int. Pharmacodyn. Ther. 214, 68–74. 1156026

[B117] Solomon R. L. Corbit J. D. (1978). An opponent-process theory of motivation. Am. Econ. Rev. 68, 12–24.

[B118] Suzuki T. Kato H. Aoki T. Tsuda M. Narita M. Misawa M. (2000). Effects of the non-competitive NMDA receptor antagonist ketamine on morphine-induced place preference in mice. Life Sci. 67, 383–389. 10.1016/s0024-3205(00)00639-1 11003048

[B119] Taylor J. H. Landeros-Weisenberger A. Coughlin C. Mulqueen J. Johnson J. A. Gabriel D.. (2018). Ketamine for social anxiety disorder: a randomized, placebo-controlled crossover trial. Neuropsychopharmacology 43, 325–333. 10.1038/npp.2017.194 28849779 PMC5729569

[B120] Tyler M. W. Yourish H. B. Ionescu D. F. Haggarty S. J. (2017). Classics in chemical neuroscience: ketamine. ACS Chem. Neurosci. 8, 1122–1134. 10.1021/acschemneuro.7b00074 28418641

[B121] Tzschentke T. M. (2007). Measuring reward with the conditioned place preference (CPP) paradigm: update of the last decade. Addict. Biol. 12, 227–462. 10.1111/j.1369-1600.2007.00070.x 17678505

[B122] Wang J. Fang Q. Liu Z. Lu L. (2006). Region-specific effects of brain corticotropin-releasing factor receptor type 1 blockade on footshock-stress- or drug-priming-induced reinstatement of morphine conditioned place preference in rats. Psychopharmacology 185, 19–28. 10.1007/s00213-005-0262-6 16374599

[B123] Wang M. Wong A. H. Liu F. (2012). Interactions between NMDA and dopamine receptors: a potential therapeutic target. Brain Res. 1476, 154–163. 10.1016/j.brainres.2012.03.029 22472597

[B124] Weisman H. (1971). Anesthesia for pediatric ophthalmology. Ann. Ophthalmol. 3, 229–232. 5163952

[B125] Wetter D. W. Smith S. S. Kenford S. L. Jorenby D. E. Fiore M. C. Hurt R. D.. (1994). Smoking outcome expectancies: factor structure, predictive validity and discriminant validity. J. Abnorm. Psychol. 103, 801–811. 10.1037/0021-843x.103.4.801 7822583

[B126] Whitaker L. R. Degoulet M. Morikawa H. (2013). Social deprivation enhances VTA synaptic plasticity and drug-induced contextual learning. Neuron 77, 335–345. 10.1016/j.neuron.2012.11.022 23352169 PMC3559005

[B127] Wikler A. (2013). Opioid Dependence: Mechanisms and Treatment. New York, NY: Springer.

[B128] Xue Y. X. Luo Y. X. Wu P. Shi H. S. Xue L. F. Chen C.. (2012). A memory retrieval-extinction procedure to prevent drug craving and relapse. Science 336, 241–245. 10.1126/science.1215070 22499948 PMC3695463

[B129] Zanos P. Moaddel R. Morris P. J. Georgiou P. Fischell J. Elmer G. I.. (2016). NMDAR inhibition-independent antidepressant actions of ketamine metabolites. Nature 533, 481–486. 10.1038/nature17998 27144355 PMC4922311

[B130] Zanos P. Moaddel R. Morris P. J. Riggs L. M. Highland J. N. Georgiou P.. (2018). Ketamine and ketamine metabolite pharmacology: insights into therapeutic mechanisms. Pharmacol. Rev. 70, 621–660. 10.1124/pr.117.015198 29945898 PMC6020109

[B131] Zarate C. A. Jr. Brutsche N. Laje G. Luckenbaugh D. A. Venkata S. L. Ramamoorthy A.. (2012). Relationship of ketamine’s plasma metabolites with response, diagnosis and side effects in major depression. Biol. Psychiatry 72, 331–338. 10.1016/j.biopsych.2012.03.004 22516044 PMC3442255

[B132] Zarate C. A. Jr. Singh J. B. Carlson P. J. Brutsche N. E. Ameli R. Luckenbaugh D. A.. (2006). A randomized trial of an N-methyl-D-aspartate antagonist in treatment-resistant major depression. Arch. Gen. Psychiatry 63, 856–864. 10.1001/archpsyc.63.8.856 16894061

[B133] Zhai H. Wu P. Chen S. Li F. Liu Y. Lu L. (2008). Effects of scopolamine and ketamine on reconsolidation of morphine conditioned place preference in rats. Behav. Pharmacol. 19, 211–216. 10.1097/fbp.0b013e3282fe88a0 18469538

[B134] Zhang L.-M. Zhou W.-W. Ji Y.-J. Li Y. Zhao N. Chen H.-X.. (2015). Anxiolytic effects of ketamine in animal models of posttraumatic stress disorder. Psychopharmacology 232, 663–672. 10.1007/s00213-014-3697-9 25231918

[B135] Zinser M. C. Baker T. B. Sherman J. E. Cannon D. S. (1992). Relation between self-reported affect and drug urges and cravings in continuing and withdrawing smokers. J. Abnorm. Psychol. 101, 617–629. 10.1037/0021-843x.101.4.617 1430600

