# Supplementary figures and images for: Ketamine Blocks Morphine-Induced Conditioned Place Preference and Anxiety-Like Behaviors in Mice
Source: Front Behav Neurosci. 2020 May 21;14:75. doi: 10.3389/fnbeh.2020.00075 (PMC7253643; doi:10.3389/fnbeh.2020.00075)

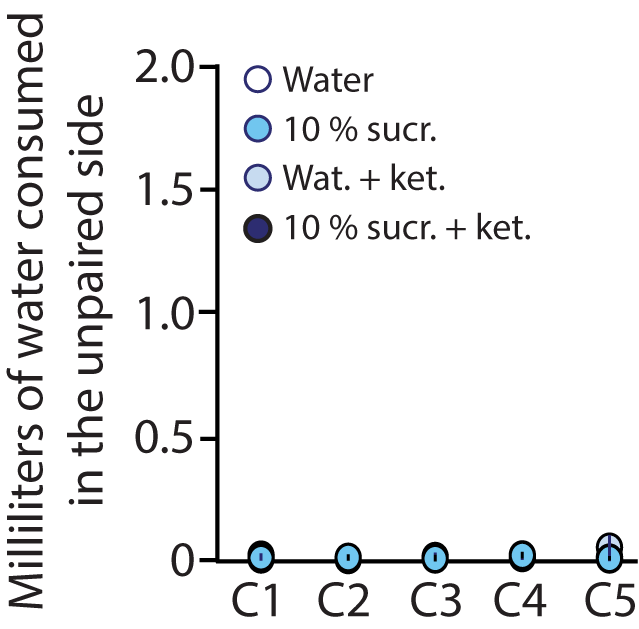

Supplement: FIGURE S1 — A summary showing that there is no significant difference in the amount of water consumed in the most preferred side among all groups (F(12,140) = 0.596, p = 0.843, two-way repeated-measures ANOVA). [file Image_1.TIF]

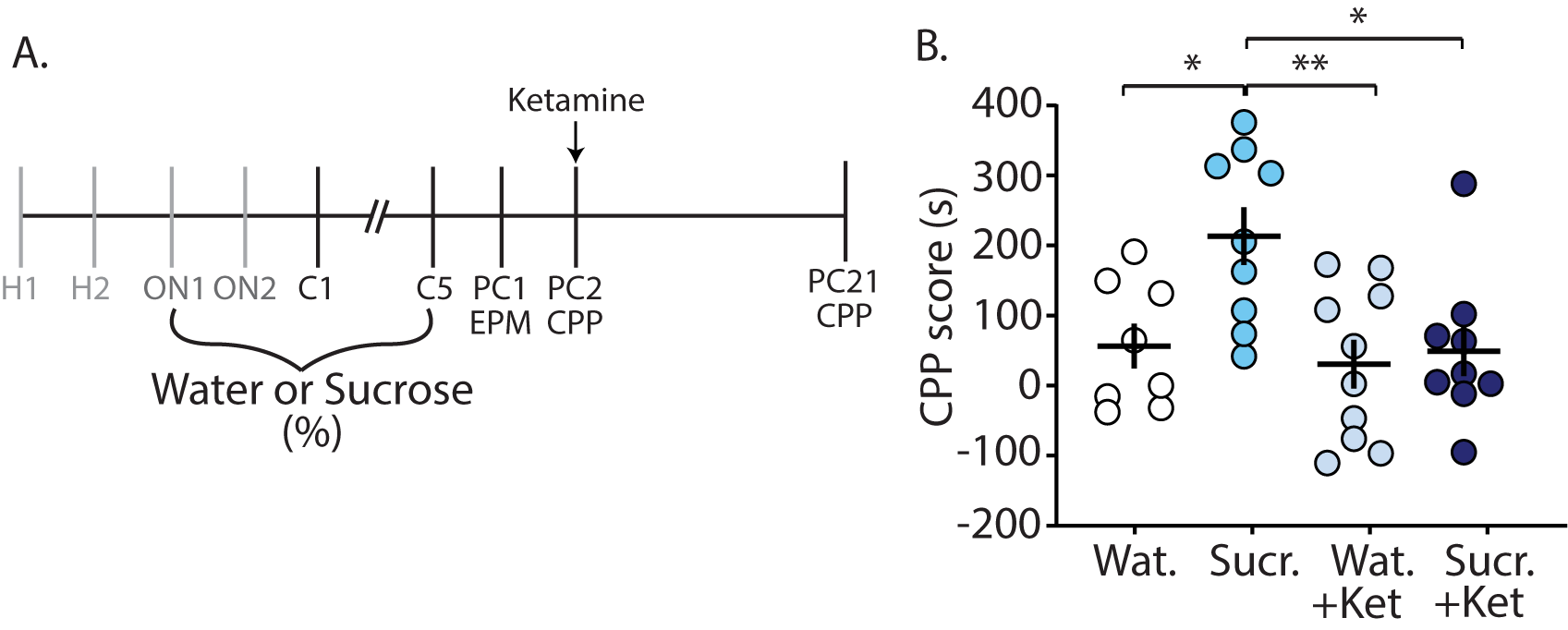

Supplement: FIGURE S2 — (R,S)-ketamine administration during early abstinence blocks the prolonged retention of sucrose-induced CPP at post conditioning day 21. (A) Time line and drug regimen of the behavioral procedure. (R,S)-ketamine (10 mg/kg, i.p.) was injected 30 min prior to the first CPP test on post conditioning day 2 (PC2). (B) Summary showing that oral self-administration of sucrose produced CPP for the sucrose-paired context 21 days after conditioning. This prolonged expression of sucrose-induced CPP was blocked by (R,S)-ketamine when injected 30 min prior to testing on PC2 (F(3,32) = 5.51, p = 0.004, one-way ANOVA, Bonferroni post hoc test). *p < 0.05, **p < 0.01. [file Image_2.TIF]
